# Virtual Surgical Planning in Orthognathic Surgery

**Published:** 2017-01-09

**Authors:** Suraj Jaisinghani, Nicholas S. Adams, Robert J. Mann, John W. Polley, John A. Girotto

**Affiliations:** ^a^Chicago Medical School at Rosalind Franklin University; Chicago, Ill; ^b^Michigan State University, Grand Rapids, Mich; ^c^Grand Rapids Medical Education Partners, Plastic and Reconstructive Surgery Residency, Grand Rapids, Mich; ^d^Pediatric Plastic and Craniofacial Surgery, Helen DeVos Children's Hospital, Grand Rapids, Mich

**Keywords:** virtual surgical planning, orthognathic, CAD/CAM, Le Fort, bilateral sagittal split

## DESCRIPTION

**Figure 1:**
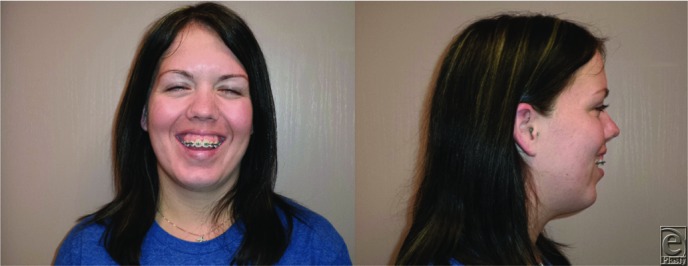
Preoperative anteroposterior and lateral photographs. Note the long midface and excessive dental and gingival show.

**Figure 2:**
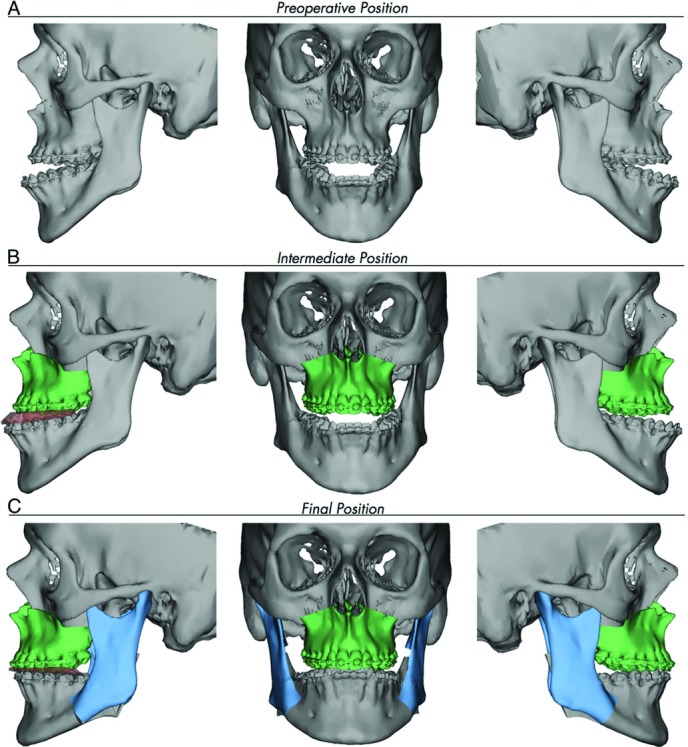
Virtual planning work flow. (a) Preoperative 3D reconstruction demonstrating excessive maxillary height and anterior open bite. (b) Planned intermediate position of the maxilla after performing a Le Fort I osteotomy with impaction and using a prefabricated splint to move the maxilla on the basis of the current position of the mandible. (c) Planned final position of the mandible after performing bilateral sagittal split osteotomies and using a second splint to move the mandible on the basis of the new position of the maxilla.

**Figure 3:**
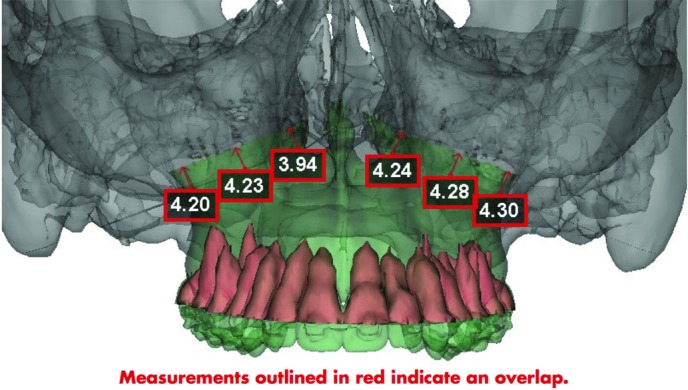
Computed tomographic scan with 3-dimensional reconstruction view of planned Le Fort I impaction. The numbers indicate movement of the maxilla in the superior vector (millimeters).

An 18-year-old woman presented for evaluation of her dental occlusion. She presented with “long face syndrome” with excessive midface height, excessive dental show in repose, and malocclusion with anterior open bite (Fig 1). She was scheduled for an impaction LeFort I osteotomy with bilateral sagittal split osteotomies. Virtual surgical planning (VSP) was utilized for operative planning (Figs 2 and 3).

## QUESTIONS

1. **What is VSP?**
2. **What are the applications of VSP?**
3. **What are the key stages of computer-assisted craniofacial surgery?**
4. **What are the advantages and disadvantages of VSP?**

## DISCUSSION

Craniofacial surgery poses various challenges due to inconsistent 3-dimensional (3D) anatomy and inadequate imaging modalities. VSP and computer-aided design (CAD)/computer-aided manufacturing (CAM) software is increasingly being used in complex craniomaxillofacial reconstruction. Benefits include increased accuracy for orthognathic surgery, reduced operating room (OR) time, and increased patient satisfaction.^1^ Surgeons collaborate with biomedical engineers via a teleconference to plan the surgery preoperatively. With the use of CAD/CAM software, the team can make various measurements and changes to the patient's 3D craniofacial skeletal anatomy and account for yaw, pitch, and roll. Using CAD software, the resections and reconstruction are virtually planned using specific osteotomy locations with precision to the 1/100th of a millimeter.^2^ At this time, bones can also be precisely measured, moved, and grafted from other body locations. Using CAM software, the team can manufacture surgical splints, osteotomy cutting guides, and plate-bending templates via 3D printing that are used during the time of surgery.

Applications of VSP include craniomaxillofacial reconstruction, temporomandibular joint (TMJ) reconstruction, trauma, and oncological reconstruction, to name a few. In craniomaxillofacial surgery, VSP-CAD/CAM technology allows surgeons to virtually perform Le Fort and mandibular osteotomies for craniofacial anomalies and maxillofacial deficiencies. Here we can advance, rotate, and resect bone tissue. This technology has been used in TMJ reconstruction taking it from a 2-stage procedure to a single-stage procedure. In the past, gap arthroplasty was done in the first stage, followed by imaging to plan implant design, followed by a second procedure to inset the TMJ implant. VSP allows for single-stage reconstruction as gap arthroplasty and implant fabrication can be conducted virtually and the implant can be produced on the basis of simulated arthroplasty.^1^ In traumatic facial injuries, VSP helps facilitate intraoperative reduction and repair with exquisite precision. CAD/CAM also allows the production of occlusal splints to achieve accurate dental relationships and facial symmetry.^3^ In the field of oncology, reconstruction of the maxilla and mandible is often limited by the lack of preoperative information regarding recipient site, graft size, cancer margins, and resection size. VSP-CAD/CAM helps resolve these issues by conducting a whole-body 3D reconstruction and performing the cancer resections with fibula free-flap reconstruction virtually.^4^

Computer-assisted craniofacial surgery involves 4 key stages: planning, modeling, surgery, and evaluation.^1^ In the planning phase, a computed tomographic (CT) scan with 3D reconstruction of the patient is conducted and sent to the design company to be applied to its CAD software. The reconstructive surgeon has a Web teleconference with biomedical engineers and together they virtually plan and conduct the surgery by performing resections, osteotomies, moving bony tissue in specific vectors, and placing bone grafts as needed (Figs 2 and 3). In the modeling stage using CAM, stereolithographic models, cutting guides, and prebent plates are produced on the basis of the virtual surgical plan and shipped to the surgeon. The next stage is the surgical phase. Virtual surgery, templates, and prefabricated implants lead to decrease OR time, increased precision, and, in the case of free-flap reconstruction, lower flap ischemia time.^5^ Finally, in the evaluation phase, a CT scan with 3D reconstruction is performed and the CAD model is overlaid with the preoperative model to obtain an objective measurement of the outcome compared with the virtual surgical plan.

Using VSP-CAD/CAM technology brings forth advantages including increased dental relationship accuracy, reduced OR time, increased patient satisfaction, and decrease cost.^5^^,^^6^ Simulating surgery preoperatively allows measurements to the 1/100th of a millimeter and when combined with 3D printed splints and customized prebent plates, the reconstructive and aesthetic outcome is superior to traditional 2-dimensional (2D) modeling and cephalometric tracing. Reduced OR time is realized with prefabricated splints, single-stage procedures (as seen in TMJ reconstruction), and having already simulated the surgery preoperatively. This decreased OR time also directly translates to reduced time under anesthesia and decreased overall cost. In 2012, using patient satisfaction surveys, subjective evaluation of functional and aesthetic outcomes were measured and compared with traditional surgery.^6^ Results showed that patients who underwent VSP reported more favorable scores than those who underwent traditional surgery. Finally, a study in 2016 showed that VSP is associated with decreased cost and OR time when compared with standard orthognathic surgery.^5^ The main disadvantage is adapting to new technology and changing the way earlier generation of surgeons perform orthognathic surgery.

**Figure 4:**
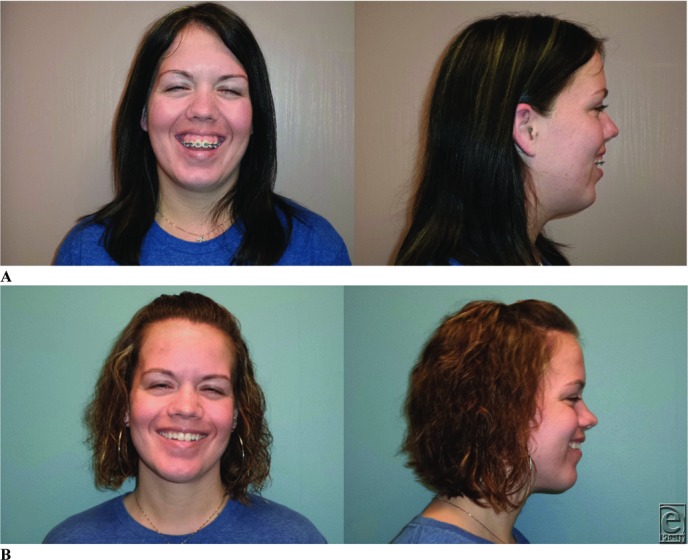
(a) Preoperative and (b) postoperative anteroposterior and lateral photographs. Note the improved occlusion, dental show, and facial profile.

VSP-CAD/CAM technology is becoming the future of craniomaxillofacial surgery with a wide variety of applications. The benefits show that this technology is superior to traditional 2D cephalometric surgery with regard to outcome (Fig 4) and cost.

## References

[1] Zielinski E, Jacobs RJ, Barker E, Rodby K, Antony AK, Motamedi MHK (2015). Virtual surgical planning in craniomaxillofacial reconstruction. A Textbook of Advanced Oral and Maxillofacial Surgery.

[2] Roser SM, Ramachandra S, Blair H (2010). The accuracy of virtual surgical planning in free fibula mandibular reconstruction: comparison of planned and final results. J Oral Maxillofac Surg.

[3] Farrell BB, Franco PB, Tucker MR (2014). Virtual surgical planning in orthognathic surgery. Oral Maxillofac Surg Clin North Am.

[4] Wang Y, Fan S, Zhang H, Lin Z, Ye J, Li J (2016). Virtual surgical planning in precise maxillary reconstruction with vascularized fibular graft after tumor ablation. J Oral Maxillofac Surg.

[5] Resnick CM, Inverso G, Wrzosek M, Padwa BL, Kaban LB, Peacock ZS (2016). Is there a difference in cost between standard and virtual surgical planning for orthognathic surgery?. J Oral Maxillofac Surg.

[6] Modabber A, Legros C, Rana M, Gerressen M, Riediger D, Ghassemi A (2012). Evaluation of computer-assisted jaw reconstruction with free vascularized fibular flap compared to conventional surgery: a clinical pilot study. Int J Med Robot.

